# Ocean currents modify the coupling between climate change and biogeographical shifts

**DOI:** 10.1038/s41598-017-01309-y

**Published:** 2017-05-02

**Authors:** J. García Molinos, M. T. Burrows, E. S. Poloczanska

**Affiliations:** 1 Arctic Research Center, Hokkaido University, Kita-21 Nishi-11 Kita-ku, Sapporo Hokkaido 001-0021 Japan; 2 Global Station for Arctic Research, Global Institution for Collaborative Research and Education, Hokkaido University, Hokkaido, Sapporo Japan; 3 Scottish Association for Marine Science, Scottish Marine Institute, Dunbeg, Oban, Argyll, PA37 1QA UK; 4 IPCC WGII Technical Support Unit, Division Biosciences/Integrative Ecophysiology, Alfred Wegener Institute, Helmholtz Centre for Polar and Marine Research, Am Handelshafen 12, Bremerhaven, 27570 Germany

## Abstract

Biogeographical shifts are a ubiquitous global response to climate change. However, observed shifts across taxa and geographical locations are highly variable and only partially attributable to climatic conditions. Such variable outcomes result from the interaction between local climatic changes and other abiotic and biotic factors operating across species ranges. Among them, external directional forces such as ocean and air currents influence the dispersal of nearly all marine and many terrestrial organisms. Here, using a global meta-dataset of observed range shifts of marine species, we show that incorporating directional agreement between flow and climate significantly increases the proportion of explained variance. We propose a simple metric that measures the degrees of directional agreement of ocean (or air) currents with thermal gradients and considers the effects of directional forces in predictions of climate-driven range shifts. Ocean flows are found to both facilitate and hinder shifts depending on their directional agreement with spatial gradients of temperature. Further, effects are shaped by the locations of shifts in the range (trailing, leading or centroid) and taxonomic identity of species. These results support the global effects of climatic changes on distribution shifts and stress the importance of framing climate expectations in reference to other non-climatic interacting factors.

## Introduction

Biogeographical shifts are some of the global responses to climate change most frequently reported in reference to terrestrial and marine life^1, 2^. Shifts in the distribution of species can alter biodiversity patterns, produce trophic and resource mismatches, spur novel biotic interactions, and initiate significant changes to the structures and functioning of ecosystems^1, 3^. These effects, which are expected to be enhanced with future climate changes^4, 5^, can have serious economic, social and human health implications^6^. From a climatic point of view, range dynamics are primarily governed by interactions between changes in climatic conditions and the physiological tolerance of a given species^7^. Under warming climatic conditions, the *a priori* expectation is for species to shift their distributions towards cooler environments at higher latitudes, in deeper waters or at higher terrain. Evidence accumulated over the past several decades demonstrate that such responses are unequivocal overall, and yet expectations based on climate alone fall short of explaining the variability in shift responses observed both across and among taxa at different geographical locations^1, 2, 8^. Accounting for this large unexplained variation is thus crucial for better anticipating and managing the effects of a rapidly changing climate.

**Figure 1:**
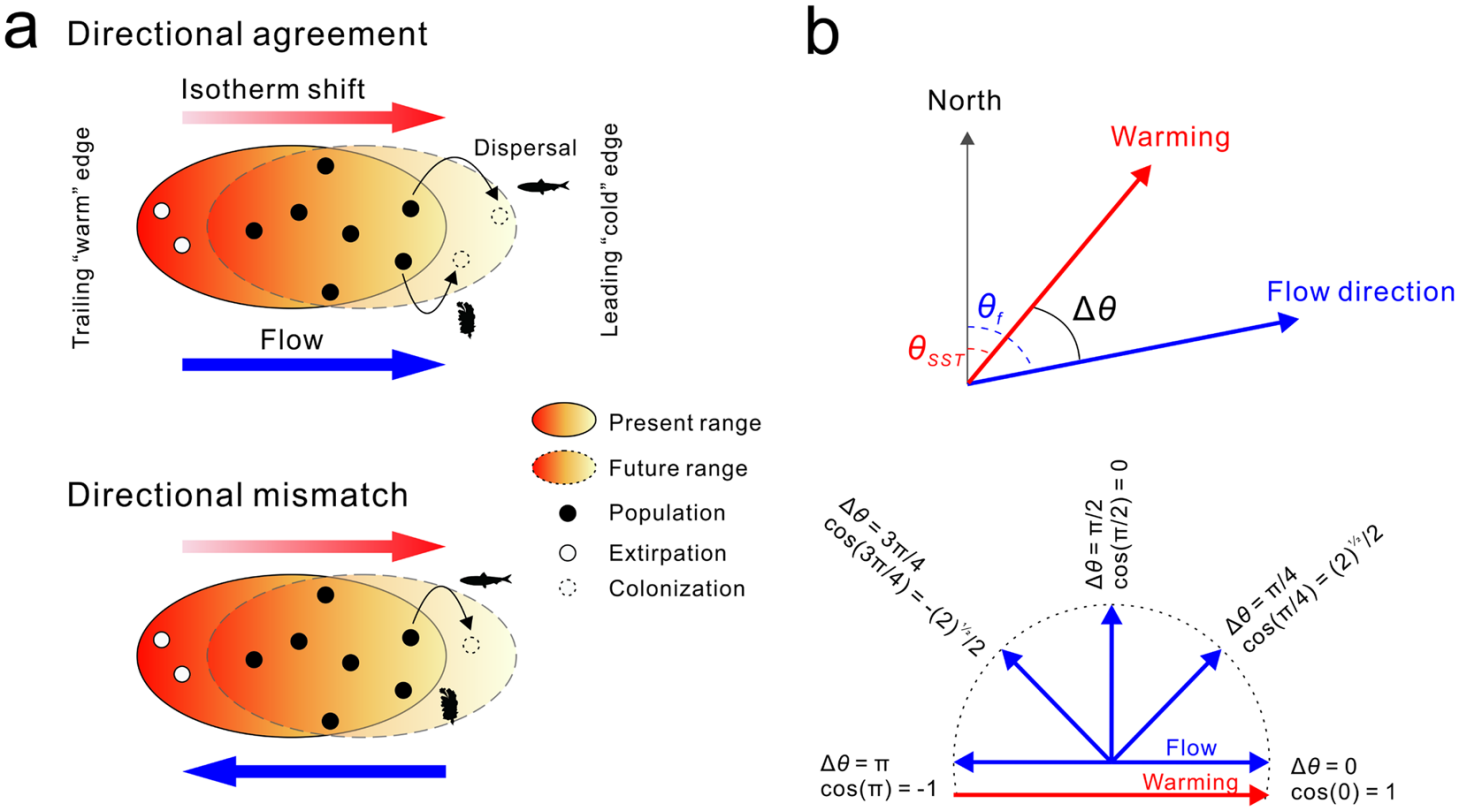
(**a**) Schematic of the hypothesized effect of directional agreement between ocean currents and thermal gradients on range edge dynamics. At the leading “cold” edge of the distribution, new habitats beyond the original range become climatically suited for the species as the species’ thermal envelope expands under warming conditions. Colonization and settlement into these habitats is dependent on the dispersal capacity of the species, which is expected to be enhanced/restricted when ocean currents correspond/do not correspond with the direction of warming. Species with active dispersal capacities (e.g., fish) and lengthy larval dispersal periods (e.g., fish/holoplankton) would be better suited to exploiting favourable flow-warming conditions than those with sessile or sedentary adults and shorter larval dispersal windows (e.g., benthic invertebrates/algae). Similarly, under intensifying levels of directional mismatching, adult active dispersion (e.g., fish) should buffer the negative effects of the current direction on thermal tracking. At the trailing edge of the distribution, warming changes climate conditions within a species’ range beyond its tolerance threshold, resulting in the extirpation of local populations and in the subsequent contraction of the range. This process is expected to occur largely independently of directional agreement. (**b**) We define directional agreement as the cosine of the difference in angle between temperature gradients and ocean currents. The resulting index ranges from −1, where both currents and isotherm movement directions are opposite, to 1, for complete directional agreement.

This heterogeneity in shift responses has been mechanistically attributed to complex interactions between climatic and other environmental and biological processes^9^, to effects of species life histories^10^, and to species-specific exposure and sensitivity to variations in local climatic conditions^11, 12^. For the former, external directional forces influencing species dispersal, such as air and water currents, have been greatly overlooked thus far despite their obvious importance^13^. For a species tracking a shifting climate, directional forces should facilitate or limit redistribution patterns depending on their directional alignment with spatial gradients of climate change (Fig. 1a). The relative importance of this factor should be concomitant with the taxonomic identity of the species involved both in terms of the capacity (from highly motile to sessile) and type (active versus passive dispersers) of response to warming. Further, given the different processes involved^14^, the effects of flow directionality on climate-driven shift responses should also be specific to the location of a given shift within a species range. For example, at the leading “cold” edge of the distribution, opportunities for range expansions arise when new habitats become climatically available beyond the current range of the species. Range expansion require the colonization of such habitats as organisms disperse from local range edge populations, a process that should be facilitated under increasing directional agreement (Fig. 1b) between ocean currents and spatial temperature gradients^15^. We expect this effect to be stronger for species with active dispersal (fish) and/or prolonged planktonic life stages (holoplankton/fish) than species with shorter larval stages and sessile or sedentary adult forms (benthic invertebrates and algae; Fig. 1a). Similarly, active dispersal should confer some capacity to counter the effects of directional mismatching. At the other extreme of the range, contractions of trailing “warm” edges are triggered by temperatures moving beyond the thermal tolerance limit of existing populations. We therefore expect contraction rates to be primarily dependent on the magnitude or rate of warming, as described by the velocity of climate change^16^, but insensitive overall to directional agreement. An analysis simultaneously evaluating the mediating effects of all these factors would provide much novel insight into the mechanisms governing climate-related shifts in species distributions^17^.

**Figure 2:**
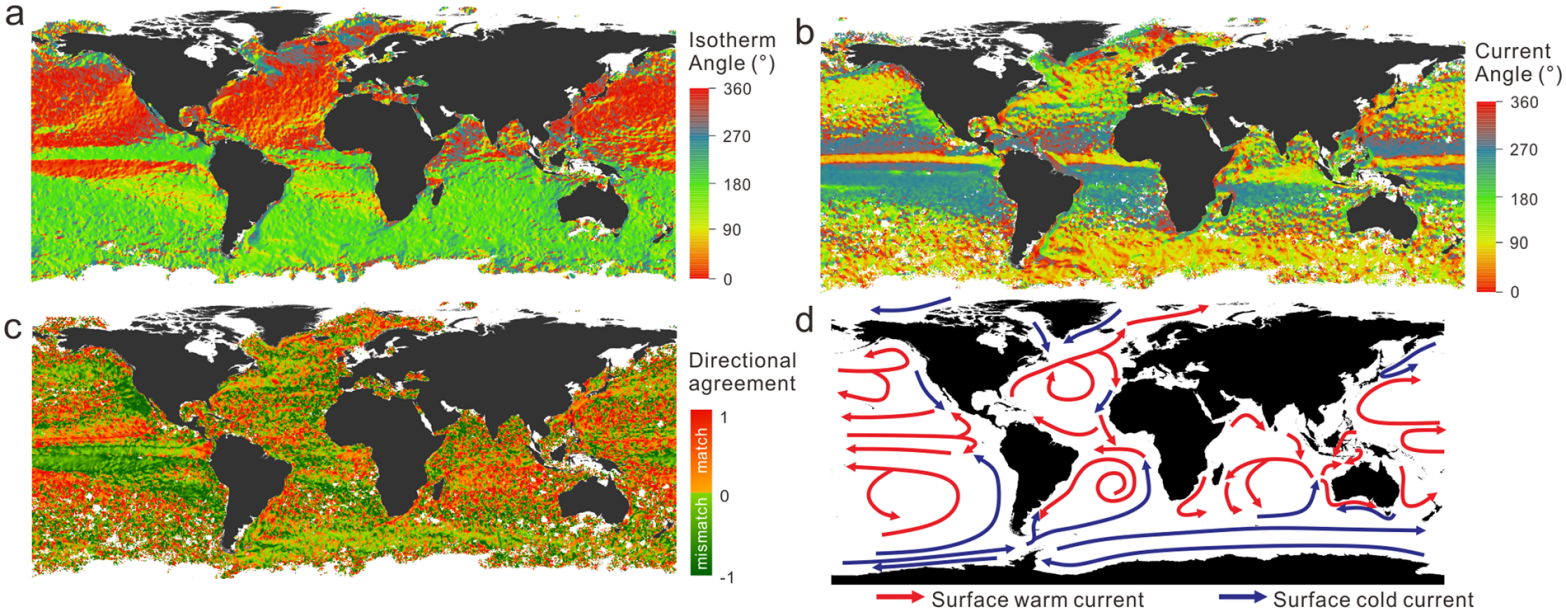
Angles and resulting directional agreement between ocean surface currents and sea surface temperature gradients. Vector angles associated to (**a**) sea surface temperature gradients and (**b**) annual mean eastward and northward current flow speed components retrieved from ocean satellite-tracked drifter data (1979–2012). (**c**) The directional agreement between both parameters estimated as the cosine of the angle difference. (**d**) A non-exhaustive schematic of major surface current systems is provided for comparison. Note how prevailing northward-flowing currents in the Northern Hemisphere and southward-flowing currents in the Southern Hemisphere (e.g., Kuroshio and Brazil currents) are dominated by red colours denoting good directional agreement, whereas green colours predominate for southward flowing currents in the Northern Hemisphere and for northern flowing currents in the Southern Hemisphere (e.g., California and Humboldt currents), denoting directional mismatching. This figure was generated using ArcGIS 10.2 (ESRI, Redland, CA; www.esri.com) and R 3.2.3 (http://www.R-project.org).

Here, we used an updated^3^ version of a meta-database recently used^2^ to evaluate the global imprint of climate change on distributional changes in marine life that accounts for a total of 270 range shifts reported in the literature where climate change was proposed as a driver of the shift (Supplementary Fig. S1 and Table S1). The dataset spans multiple taxonomic groups and types of responses (i.e., shifts in population centroid and leading and trailing edges). Using generalized linear models, we employed a model selection approach^18^ to predict observed distribution shifts resulting from (see Methods, Table 1) (i) the changes in local climate conditions using the velocity of climate change^15^, (ii) the taxonomic identity of the species involved, (iii) the directional agreement between spatial gradients in sea surface temperature (SST) and ocean currents, and (iv) the location of a given shift within a species range. To define local directional agreement between spatial thermal gradients and ocean currents, we propose the use of the cosine of the difference in angles associated with both parameters (see Methods). This new, simple metric generates an index that ranges from −1 (for ocean currents in the opposite direction to spatial thermal gradients) to 1 (for currents and gradients in the same direction) (Figs 1b and 2). Both the questions being addressed and the processes involved are directly transferable to freshwater species and all terrestrial organisms relying on aerial dispersal processes, including many plants, insects, and birds.

**Table 1:** Description of the two categorical predictor variables used to predict observed distribution shifts.

**Group**	**Early development**	**Adult**
**Taxonomic identity**		
Fish	Lecithotrophic larvae	Actively dispersing, high mobility
Benthic invertebrates	Planktotrophic larvae or non-pelagic development	Sessile or sedentary
Benthic macroalgae	Planktonic propagules	Sessile
Holoplankton	Planktonic with high passive drift potential	
**Shift location**		
Location	Relation to warming	Governing process
Leading edge	Cold/high-latitude extreme	Range expansions beyond range limits in response to the development of new climatically suitable habitats
Trailing edge	Warm/low-latitude extreme	Range contractions within range limits in response to climatic conditions surpassing thermal tolerance
Distribution centroid	Whole range	Compound

## Results and Discussion

We found unconditional support for the full model, including support for the effect of climate velocity and for the three-way interaction among directional agreement, biological identity and shift location, which accounts for 66% of the total variance in observed shifts; this is over 25% more than the variance explained by a model based on the climate effects alone (Table 2a). The rate of warming expressed as the velocity of climate change nevertheless has a highly statistically significant positive effect on shift rates (*β* = 0.21, *t* = 6.92, *p*-value = 3.9 × 10^−11^; Table S2) and accounts for nearly two thirds of the total variance explained by the full model (40.4%; Table 2), confirming the previously reported global effects of warming on range shifts of marine biota^2^. However, the effect of warming on shift responses does not concern only how fast climatic conditions are changing but also to what extent the direction of warming matches that of ocean flows, an effect modulated both by the location of a shift within a species range and by the taxonomic identity of the species involved (20.02 decline in deviance from the full model to a model without the 3-way interaction, *F*_253,249_ = 5.94, *p*-value = 1.4 × 10^−4^).

**Table 2:** Comparison of the climate-expectation Gamma (log-linked) GLMs for the prediction of shift responses (*n* = 270) ranked in terms of AICc values (for information on the model coefficients, see Table S2).

Model	M_1_ (Full model)	M_2_	M_3_	M_4_	M_climate_
Climate velocity	+	+	+	+	+
Directional Agreement (DA)	+	+	+	+	
Shift Location (SL)	+	+	+	+	
Biological Identity (BI)	+	+	+	+	
DA × SL		+	+	+	
DA × BI		+		+	
BI × SL		+	+		
DA × BI × SL	+				
AICc	6446.1	6898.8	7072	7306.1	9928.9
∆AICc	−	452.72	625.96	860.02	3482.8
Weight	1	<0.001	<0.001	<0.001	<0.001
D-squared	0.66	0.636	0.629	0.614	0.404

Models fit the fourth-root transformed observed shifts (km/decade) weighted by the number of observed years per observation on the fourth-root transformed climate velocity estimate (km/year) and the three-way interaction among biological identity, shift location, and directional agreement between thermal gradients and current flows. Selection was based on the Akaike Information Criterion corrected for finite sample sizes (AICc). The D-squared statistic denotes the proportion of total variance explained by the model and has an analogous interpretation to that of the coefficient of determination used in linear regression models. The results are presented for the first 4 ranked models and the climate-expectation model (i.e., using only climate velocity as a predictor).

**Figure 3:**
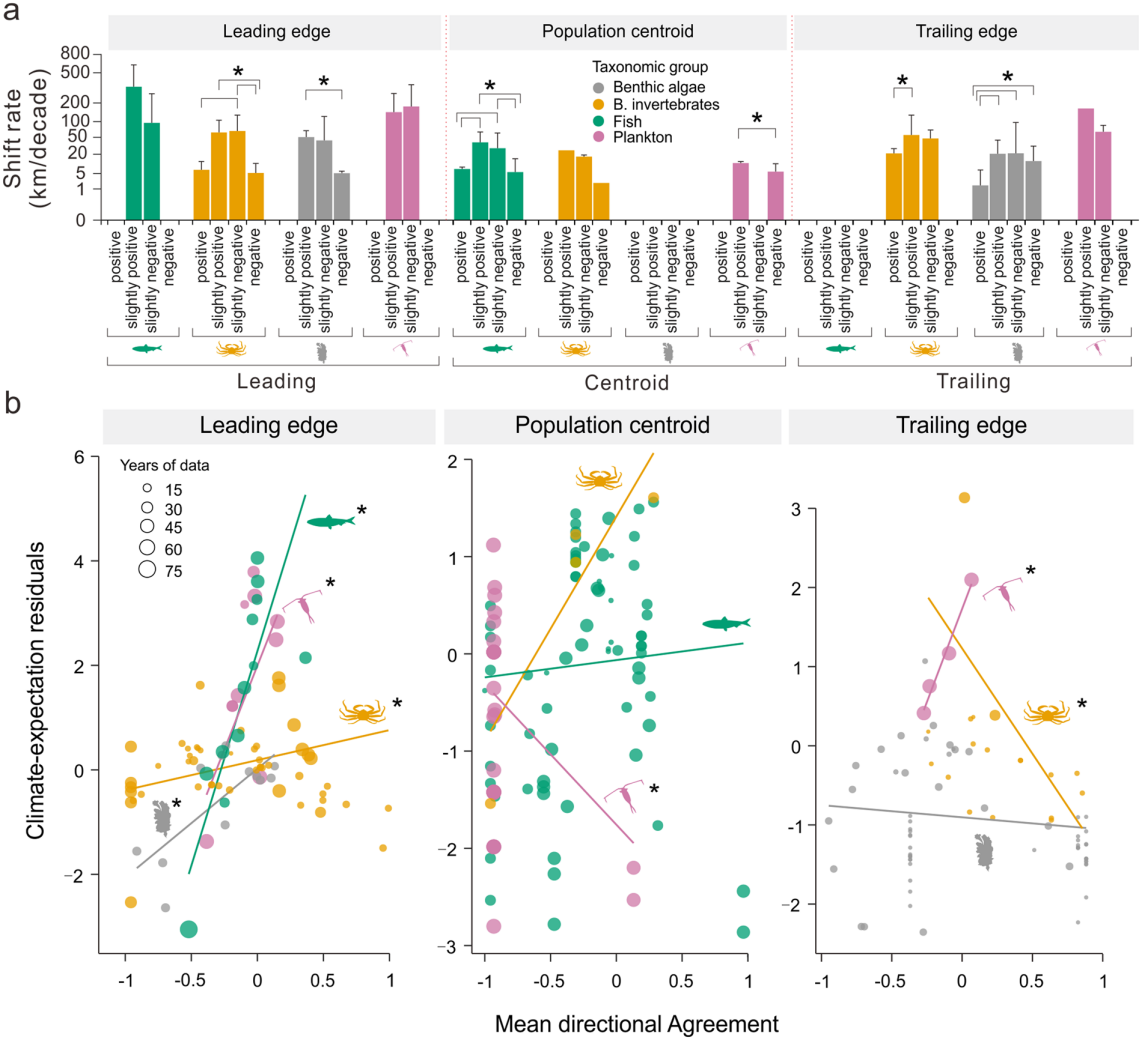
Effect of three-way interactions between directional agreement, taxonomic identity and the location of the shift within the species range (leading, centroid or trailing). (**a**) Mean observed shift rate (+1 standard deviation) weighted by the number of observed years per shift record and grouped by the three parameters. Directional agreement was categorized into four groups reflecting the strength of the agreement between currents and thermal gradients (cos(π/4) = 0.71; see Fig. 1b): negative [−1, −0.71], slightly negative (−0.71, 0], slightly positive (0, 0.71), and positive [0.71, 1]. Pairwise statistically significant differences between agreement categories within each location-taxa combination are denoted by an asterisk (two sample *t*-tests, *α* = 0.05). Levels not represented are those for which no data were available. (**b**) Scatterplots of residuals from the climate-expectation model against mean directional agreement by taxonomic group for leading edges, range centroids and trailing edges. Positive/negative residuals suggest observed responses ahead of/lagging behind the mean expected climate response. Lines correspond to the resulting fitted linear regressions weighted by the number of years per observation, with asterisks denoting the statistical significance of slopes (*α* = 0.05).

Expansion rates at the leading edge of the range decreased significantly under increasing directional mismatching between ocean currents and thermal gradients for benthic invertebrates and algae (Fig. 3a), supporting our initial expectation concerning dispersion-mediated effects of flow directionality on climate-driven range expansion (Fig. 1a). On the other hand, we found no evidence that active dispersion offsets the negative effects of mismatched directionality for fish compared to passive dispersing taxa, though our lack of observations for the most extreme directional mismatch category for fish limits our inference. Nevertheless, and though flow-dependency is arguably most important for the redistribution of sessile species relying on the passive dispersion of larvae and propagules^19^, highly motile taxa such as fish with active-dispersing life stages, or holoplankton which are permanent members of the plankton, should be better equipped to exploit the favourable conditions provided by flows when matching thermal gradients^20^. The fact that fish and plankton showed consistently faster expansion rates than benthic organisms for the same level of directional agreement supports this hypothesis (Fig. 3a), reflecting the importance of life dispersal-related history traits for range expansion.

At the trailing edge of the range, we found indications of an opposite effect of flow directionality on climate-driven range contractions, with benthic organisms (algae and invertebrates) contracting their ranges significantly slower under increasing directional agreement (Fig. 3a). This result contradicts our initial hypothesis, as range contractions were expected to operate independently of directional agreement through the effects of climate on local populations via decreased fitness, increased mortality and ultimate extirpation as climate conditions approach and surpass physiological thresholds^14^. However, the physiological tolerance of local populations within a species range can vary widely due to adaptation and acclimatization to local environmental conditions^7, 21^. External directional forces such as ocean currents and air flows could then contribute to climate-driven extirpation dynamics by shaping how local populations are connected throughout the range of a given species^22, 23^. Contraction rates under directional agreement may therefore be slowed through enhanced adaptive evolution to warming in downstream populations within the distribution range, which is promoted by increasing genetic variations from the arrival of individuals from populations at the trailing edge that are already experiencing climatic conditions that will exist at higher latitudes in the future^24, 25^. On the other hand, under directional mismatching, the situation is reversed, with upstream populations swamping trailing edge populations with maladaptive gene flows^17, 18^, thus facilitating increased contraction rates.

With the exception of contraction events triggered by extreme climatic events^26^, climate-related range contractions are often regarded as an equally frequent^27^ but slower^2, 14, 28^ processes than expansions. Setting aside potentially confounding effects introduced by differences in detectability associated with range expansions and contractions^14^, the immediate effects of such discrepancies would involve an increase in the overall species range under warming conditions and the development of a biodiversity surplus as immigration rates exceed those of extinction at specific localities^5^. Our results point to a directional alignment between ocean flow direction and thermal gradients as a possible mechanism that may enhance such conditions, where increasing directional agreement accelerates expansion rates and delays contractions, particularly for organisms relying on the passive dispersal of planktonic eggs and larvae. Such effects could, however, be transient if reduced contraction rates signal a delayed response to warming (i.e., accrual of climatic debts)^29^, reversing into a future net loss of biodiversity once such a response is triggered^30^. An examination of the residuals from the climate-expectation model, used as a proxy for how closely species track changes in thermal conditions (see Methods), suggests that this may be the case for benthic organisms, for which trailing edges contract behind climatic conditions with increased lags (i.e., larger negative residuals) under increasing directional mismatching (Fig. 3b). On the other hand, conditions observed for these taxa under increasing levels of directional mismatching, i.e., higher contraction and lower expansion rates, are linked to a build-up of immigration lags that, if not offset in the future, can further exacerbate climate change risks. These are likely to be enhanced through habitat selectivity, with organisms with specific substrate and habitat requirements, like many benthic taxa, being more limited in their expansion to new favourable environments than generalist groups such as fish and plankton^31^, therefore making them more likely to develop immigration lags.

Unlike the distribution limits, no clear pattern emerged among taxonomic groups from the effects of directional agreement on shift rates at range centroids or mismatching between observed centroid shifts and climate expectations (Fig. 3). Range centroid shifts capture changes occurring within the entire range of a given species and hence are often more idiosyncratic in their responses to climate change than range edges, where populations are usually at or closer to physiological tolerance limits. Similarly, higher variation in shift response introduced by the interaction of climate change with other interactive non-climatic biotic and abiotic factors is more likely over the broader geographical extent of the entire range^32^.

Our results demonstrate how a contextualization of range shifts to alignment between warming and directional forces (e.g., water or wind flow) can, in combination with other factors such as life-history attributes or particular geographical and habitat settings^33, 34^, help explain differences in expansion and extinction rates while providing mechanistic insight into the transient and net effects of climate change on biodiversity. We show how the use of a simple metric accounting for the mean directional agreement between ocean currents and spatial thermal gradients can significantly improve the amount of variance explained in observed distribution shifts over and above those accounting for isolated effects of changes in climatic patterns. It is however necessary to acknowledge that our meta-analysis does not account for the important role of fine-scale factors on range shift responses to climate change. For example, warming often exhibits heterogeneous patterns in space, season and time, lost from long-term monotonic warming signals, that may be important drivers of observed range shift responses^35^. Similarly, ocean currents are highly dynamic and can change in intensity over time^36^ as well as present periodical changes in flow paths^37^ or even reverse direction seasonally^38^. While our model captures the mean directional effect, seasonal directionality would be particularly important if species are shifting in (a)synchrony with the appropriate phase of the current seasonal cycle. If existing, such effects will be missed from our mean-effect model resulting in increased residuals and lower predictive power, but cannot be accounted for in our meta-analysis given the lack of information on the timing of the observed biological shifts. Geographical configuration^34^ and biotic interactions^39^ are also important factors. Lastly, vertical (deepening) as well as horizontal (geographic) distribution shifts are possible^40^. All these factors are important and highlight the complexity in anticipating distribution shifts in response to climate change. Our metric can nonetheless be developed in relation to more specific modelling approaches. For example, the persistence and expansion of benthic organisms in asymmetric flow is mainly stochastic and is primarily dependent on the upstream dispersal of planktonic larvae via random fluctuations in currents around the mean current directionality^22^. The capacities to exploit opportunities offered from temporal and spatial variability windows in flow conditions are dependent on different phenological and demographic adaptations regulating traits such as the timing and number of spawning events, the number of propagules released into the water column or dispersal periods^22, 41^. Parallelisms can be easily found among freshwater species or terrestrial species relying on aerial dispersal mechanisms^42^. Our metric could be easily adapted to measure the strength of the directional agreement between flow and thermal gradients during the particular spawning/dispersal season of a given species and can be used as a predictor in species distribution modelling approaches.

## Methods

### Velocity of climate change

We calculated local (1° × 1° resolution) climate velocities corresponding to the time period reported for each shift observation using mean annual sea surface temperatures (SSTs) drawn from the Hadley Centre HadISST v1.1 dataset^43^ as the ratio of the temporal linear trend to the spatial gradient in temperature. Following Burrows *et al*.^15^, temporal trends were calculated as the slope of the linear regression of mean annual SST on time (years), and spatial gradients based on the vector sum of north-south and east-west gradients were applied to each cell using a 3 × 3 neighbourhood window. Single mean velocity estimates for each shift were then obtained from all grid cell values within a circle of radius equal to the reported shift distance^2^. The velocity of climate change gives the speed and direction with which hypothetical species would need to move to remain at the same temperature experienced today at a particular location in the future^15^.

### Directional agreement between ocean flow and thermal gradients

We used ocean satellite-tracked drifter data^44^ of annual mean eastward and northward flow speed components (0.5° × 0.5°) averaged for 1979–2012 to estimate the corresponding bearing given by the resulting flow vector. SST spatial gradients and associated angles were calculated as shown in^15^ using field data collected from buoys^44^ for the same period to maintain consistency between thermal and flow data. The directional agreement between ocean flow and spatial thermal gradients was then estimated as the cosine of the angle difference between flow and SST gradients. This index ranges from −1 to 1 for opposite and matching directions, respectively, and takes a value of 0 when vectors are perpendicular (Fig. 1b). Single estimates were then calculated for each shift by averaging over all cell values within a circular buffer of radius equal to the reported shift distance.

### Distribution shifts

Shift records were distributed globally (Fig. S1) and were sourced from the recently updated^3^ meta-dataset used by Poloczanska *et al*.^2^, giving a total of 391 reported climate-driven distribution shifts from 48 published studies. Given our interest in the combined effects of warming and ocean flows, we excluded observations of biota that are not confined to the aquatic environment (i.e., sea birds and terrestrial mammals, *n* = 5). We also lost 58 observations for which ocean flow data were not available (Figs 1 and 2e), leaving a total of 327 observations, of which 57 (17.4%) were null responses. Further information on the dataset, including data extraction, quality control and processing data, are provided elsewhere^2^. For the purposes of this study, for each observation, we extracted its geographic location and duration (years), the positioning of the shift within the range (range centroid, leading or trailing edge), and the reported rate of shifting (kilometres per decade) as the absolute distance shifted^2^.

### Taxonomic identity

We grouped each observation into four general taxonomic groups (Table S1 and Fig. S1): benthic algae (*n* = 72), benthic invertebrates (e.g., crustaceans, molluscs, corals; *n* = 72), fish (bony and non-bony; *n* = 83), and plankton (phyto- and zooplankton; *n* = 43). Though grouping at a higher taxonomic resolution was possible, we decided to use these larger groups to generate comparable sample sizes, thus making comparisons among groups more meaningful while retaining overarching differences in dispersal-related traits among groups (Table 1).

### Statistical analysis

Our dataset presented some analytical challenges. First, it consisted of semi-continuous shift data with a point-mass at zero (i.e., zero inflation) and a continuous right-skewed distribution for positive values. Second, a recorded zero shift response can either be a valid observed response, “true zero” with no actual shift response, or a partial observation censored at 0 resulting from a lack of detectability arising, for example, due to a poor sampling resolution. Such combined zero responses cannot be modelled using conventional Tobit models designed for censored data or using two-part zero-inflated models, which assume that all zeros are valid observed responses^45^. Though some alternatives have been proposed as ways to model mixed zero inflated data^46^, these models are not yet sufficiently adapted for the type of analysis required here. Further, given the nature of our meta-dataset, differentiating between censored and true zeros is often not clear from a given context, further hindering the application of such methods. We therefore chose to focus exclusively on reported shift responses (*n* = 270; Table S1 and Fig. S1), thus targeting factors driving the magnitude of observed shifts rather than those triggering such responses.

We used gamma-distributed generalized linear models (GLM) with a log-link to predict the mean magnitude of shift responses. Predictors included climate velocity estimates as climate expectations, the directional agreement between warming and ocean flows, the location of the shift within the species range, and the biological identity of the species. Our initial full model included climate velocity and three-way interactions between shift locations, biological identity with directional agreement (Table 2). We applied a multi-model averaging selection approach^18^ involving all possible predictor combinations from the full global model. The model selection approach was based on the Akaike Information Criterion corrected for finite sample sizes (AICc), and the proportion of total variance explained by the model was defined using the D-squared statistic^47^, which has an analogous interpretation to the coefficient of determination in linear regression models. Unconditional support for a candidate model was given when its corresponding AICc weight was 0.95 or higher. Model diagnostics on the resulting most parsimonious model were assessed both numerically and visually through the inspection of residual patterns (see Supplemental Material). Both climate velocities and observed shifts were fourth-root transformed to improve residual patterns.

Residuals from the climate-expectation model, which reflect the isolated effects of climate on range shifts using climate velocity as the sole predictor, were used as a proxy for a species’ capacity to track shifts in thermal conditions. Positive/negative residuals denote species that are shifting ahead of/lagging behind the mean response expected from changes in thermal conditions. Regressions of residuals against mean directional agreement for each reported shift therefore provide a contrast for how flow-thermal directionality influences the capacity of a species to track shifting thermal envelopes independently of the magnitude of that shift. To do this, we conducted a preliminary assessment using alternative velocity estimates to find the climate-expectation model yielding the best fit to the observed shifts. Differences between estimates referred to (i) the temporal extent at which velocities were calculated (either specific to each shift observation or using a fixed period running from 1960–2009 as in^2^); (ii) restricting or not restricting^2^ estimates to velocities from cells neighbouring land for coastal species; (iii) using estimates based on annual mean^2^ SSTs or both annual mean and maximum/minimum monthly SSTs to better define shifts at the population centroid and trailing/leading edges, respectively; (iv) using median rather than mean velocities^2^ when averaging regionally (i.e., very high velocities can result from small changes in temperature where spatial gradients are too shallow^15^); and (v) considering all velocities over a shift distance^2^ or only those corresponding to statistically significant changes in temperature over time to better capture the true magnitude of warming species are facing within the spatial extent dictated by the shift distance. We found unconditional support (1 model weight, 21.8 AICc units above the model ranked second; Table S3) for a model based on mean-aggregated velocity estimates comprising only statistically significant velocities (non-significant velocities taken as 0) and limited to cells neighbouring land for coastal species. Models based on median-aggregated estimates did not perform better in most cases than their counterparts based on mean values, perhaps because we truncated the spatial gradients (0.001 °C/km) when computing the local climate velocities to limit the inflating effects of near-zero spatial gradients^48^. Including velocities based on observation-specific time periods or using velocities based on annual mean, minimum or maximum monthly SST depending on the location of a shift within a given range also did not improve the model’s performance. The latter result may reflect a limitation of our mean monthly SST data, which failed to represent extreme climatic conditions.

## Electronic supplementary material

Supplementary Material

Table S1
