# Supplementary material for: Ocean currents modify the coupling between climate change and biogeographical shifts
Source: Sci Rep. 2017 May 2;7:1332. doi: 10.1038/s41598-017-01309-y (PMC5431058; doi:10.1038/s41598-017-01309-y)
Supplement: Supplementary file 1 — Supplementary Material [file 41598_2017_1309_MOESM1_ESM.pdf]

Supplementary Information for

Ocean currents modify the coupling between climate change and biogeographical shifts

J. García Molinos [*], M. T. Burrows, and E. Poloczanska

1. Model diagnostics
2. Table S1. Details of the meta-data set used in this study (separated csv file).
3. Table S2. Coefficients for the resulting most parsimonious model predicting range shift rates from velocity of climate change, the interaction between location of the shift, taxonomic identity and directional agreement between ocean currents and spatial thermal gradients.
4. Table S3. Comparison of the climate-expectation Gamma (log-linked) GLMs for prediction of shift responses ranked in terms of AICc and details for the resulting most parsimonious model.
5. Table S4. Generalized variance inflation factors (GVIFs) for variables included in the global GLM.
6. Figure S1. Distribution of the shift records included in the analysis by major ocean region.
7. Figure S2. Model diagnostic plots for the most parsimonious model for the prediction of distribution shifts in marine biota.

Model diagnostics

Multicollinearity among predictor variables was assessed using generalized variance-inflation factors (GVIF)[1], which account for the existence of levels in categorical variables in the calculation of VIFs (i.e., the GVIF for a continuous variable is equivalent to the VIF). We used its linearized version ($GVIF^{[1/(2*df)]}$), which essentially puts the index on a linear scale by reference to the number of degrees of freedom of the predictor, thus making it comparable across dimensions[1]. For continuous variables (i.e., 1 degree of freedom), the linearized GVIF indicates the degree to which the standard error is increased due to multicollinearity. For categorical variables, the interpretation is similar in terms of the reduction in precision of the coefficients estimation. All predictor variables presented linearized GVIFs below the control threshold of 2, equivalent to a VIF of 4, corresponding to a tolerance of 0.25 (i.e., 75% of the predictor's variance explained by the other predictors)[2] (Table S4).

Residual patterns (Fig. S2a-b) of the most parsimonious model (Table 1) showed no particular indication of violation of model assumptions (i.e., independence, homoscedasticity and normality). Given the lumped structure of our meta-data set, where observations are spatially biased towards specific ocean regions (Fig. 1), we checked for spatial autocorrelation in the residuals of the best model. Both visual (Fig. S2c) and numerical (Mantel test for correlation between spatial and residual distance matrices $r = 0.007$, p-value = 0.35 based on 9999 permutations) analysis did not suggest, however, the existence of significant spatial autocorrelation in the residuals. The model did also not suffer from overdispersion as indicated by the rate of the Pearson χ^2 test statistic to the residual degrees of freedom[3] (209.83/249 = 0.85).

References

1. Fox, J. & Monette, G. Generalized collinearity diagnostics. *J. Amer. Statist. Assoc.* **87**, 178-183, (1992).
2. Hair, J. F., Black, W. C., Babin, B. J. & Anderson, R. E. *Multivariate Data Analysis: A Global Perspective*. 7th edn, (Prentice Hall, 2009).
3. Anderson, D. R. & White, G. C. AIC model selection in overdispersed capture-recapture data. *Ecology* **75**, 1780-1793, (1994).

Supplementary Tables

Table S1. Meta-data set used in this study (csv file). Fields include: ID (ordinal) observation identifier, New (categorical) whether a record is (1) or not (0) new relative to the data set used by Poloczanska et al. (2013), Lon/Lat (continuous) coordinates of shift observations, Sci_Name (text) scientific name of the species, Taxo_Group (text) taxonomic group used for analysis, Range_Sect (text) location of the shift within the distribution range, Obs_Shift (continuous) observed distribution shift in km/decade, Time_Span (ordinal) number of years for the shift, yrs_Data (ordinal) number of years with data, Direc_Agree (continuous bounded -1, 1) directional agreement between ocean flows and temperature gradients, VoCC (continuous) velocity of climate change in km/year, Reference (text) literature source reference.

Table S2. Coefficients for the resulting most parsimonious model predicting range shift rates from velocity of climate change, and the interaction among shift location, taxonomic identity and directional agreement between ocean currents and spatial thermal gradients. Leading edges and fish taken as contrast levels for the two categorical predictors shift location and biological identity in the interaction (absent combinations correspond to combinations for which no data was available).

**Coefficient**	**Estimate**	**Std. Error**	***t***	**P(>|*t*|)**
Intercept	1.084	0.069	15.684	**$< 2e^{-16}$**
Climate velocity	0.212	0.031	6.918	**$3.86e^{-11}$**
Directional Agreement	1.158	0.195	5.952	**$6.94e^{-05}$**
Shift Location (Centre)	-0.466	0.062	-7.504	**$1.10e^{-12}$**
Shift Location (Trailing)	-0.083	0.106	-0.784	0.43383
Taxonomic Identity (B. algae)	-0.356	0.125	-2.842	**0.00485**
Taxonomic Identity (B. invertebrate)	-0.407	0.07	-5.851	**$1.53e^{-08}$**
Taxonomic Identity (Plankton)	-0.081	0.076	-1.062	0.28920
SL (Trailing) : TI (B. algae)	-0.373	0.164	-2.271	**0.02399**
SL (Centre) : TI (B. invertebrate)	0.559	0.138	4.047	**$6.94e^{-05}$**
SL (Trailing) : TI (B. invertebrate)	0.285	0.159	1.799	0.07323
SL (Centre) : TI (Plankton)	-0.214	0.116	-1.840	0.06691
Shift Location (Centre) : DA	-1.028	0.203	-5.068	**$7.85e^{-07}$**
Shift Location (Trailing) : DA	0.005	0.575	0.008	0.99350
TI (B. algae) : DA	-0.429	0.322	-1.330	0.18460
TI (B. invertebrate) : DA	-0.956	0.212	-4.490	**$1.09e^{-05}$**
TI (Plankton) : DA	-0.423	0.34	-1.242	0.21535
SL (Trailing) : TI (B. algae) : DA	-1.022	0.637	-1.605	0.10971
SL (Centre) : TI (B. invertebrate) : DA	1.342	0.305	4.397	**$1.63e^{-05}$**
SL (Trailing) : TI (B. invertebrate) : DA	-0.881	0.66	-1.334	0.18357
SL (Centre) : TI (Plankton) : DA	0.186	0.356	0.522	0.60218

Table S3. a) Comparison of the climate-expectation Gamma (log-linked) GLMs for prediction of shift responses ranked in terms of AICc and b) details for the resulting most parsimonious model. Results are presented for the first 5 ranked models together with the model used in the previous assessment by Poloczanska et al. (2013). Models fitted on fourth-root transformed observed shifts (km/decade) using different fourth-root transformed climate velocity estimates (VoCC; km/year) weighted by the number of observed years per observation. Selection based on the Akaike Information Criterion corrected for finite sample sizes (AICc), which, in this case, corresponds to the selection of the model with lowest residual deviance given all candidate models shared number of parameters and observations. The D-squared statistic indicates the proportion of total variance explained by the model and has an analogous interpretation to that of the coefficient of determination in linear regression models.

a)

Velocity estimate*	Rank	AICc	ΔAICc	df	Weight	D-squared
VoCCsgmnD	1	9929	-	3	1	0.404
SSTsgmn	2	9950.8	21.8	3	<0.001	0.401
SSTsgmnDL	3	10098.9	169.9	3	<0.001	0.385
SSTsgmnL	4	10100.8	171.8	3	<0.001	0.385
SSTsgmnLTp	5	10553.7	624.8	3	<0.001	0.333
SSTmn	13	11925.8	1996.8	3	<0.001	0.147

b)

Coefficient	Estimate	Std. Error	*t*	P(>|*t*|)
(Intercept)	0.498	0.027	18.79	**< 0.0001**
VoCCsgmnD^(1/4)	0.344	0.024	14.2	**< 0.0001**

* sg: statistically non-significant velocities considered as 0, mn: estimates based on regional averages, D: estimates based only on values from cells neighboring land for coastal species, Tp: estimates calculated for the specific time period of each observation, L: estimates based on mean and extreme monthly temperature values depending on the location of the shift within range.

Table S4. Generalized variance inflation factors (GVIFs) for variables included in the global GLM. Multicollinearity was assessed on the linearized version of the index (right-most column) assuming a threshold of 2, equivalent to a tolerance of 0.25.

Variable	GVIF	df	$GVIF^{[1/(2*df)]}$
Climate velocity	2.4	1	1.55
Directional agreement	1.89	1	1.38
Shift location	3.11	2	1.33
Taxonomic group	3.17	3	1.21

Supplemental Figures

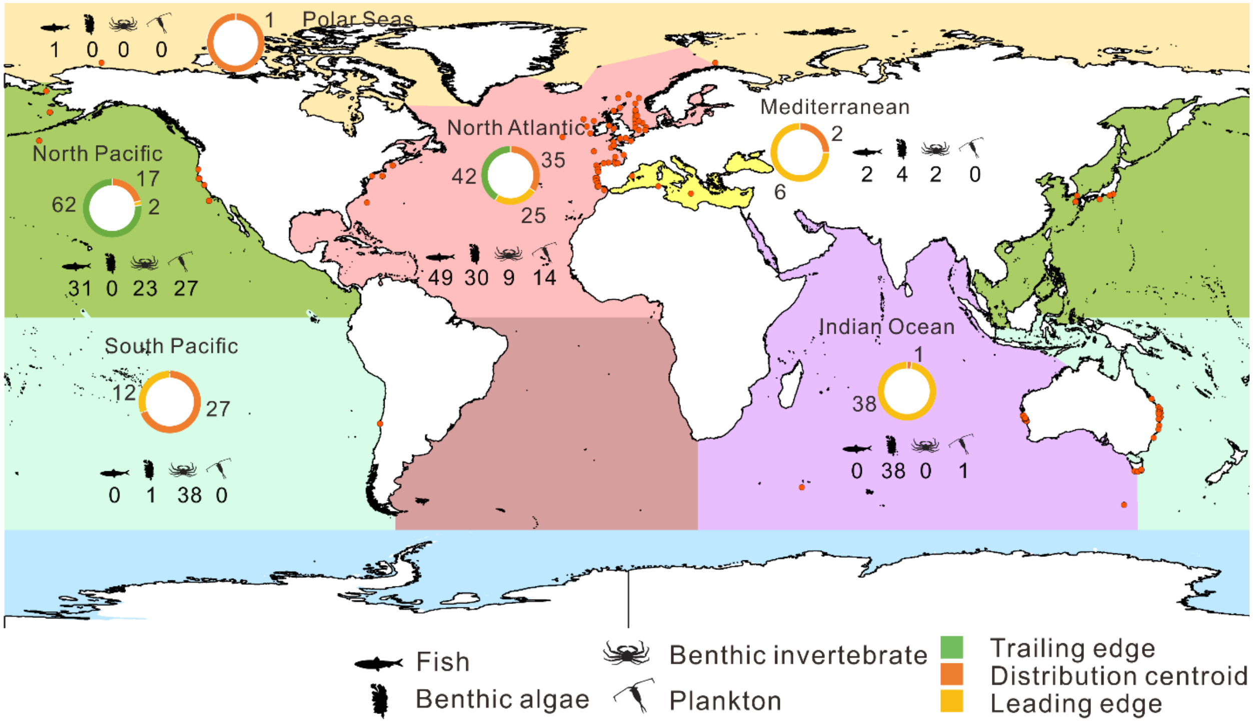

Figure S1. Distribution of the shift records included in the analysis by major ocean region. Circular plots give the number of reported shifts by location within the range of the species, and silhouettes refer to observations per taxonomic group. Geographical location of records indicated by the points (note that several records for different species might correspond to a single location). This figure was generated using ArcGIS 10.2 (ESRI, Redland, CA; www.esri.com).

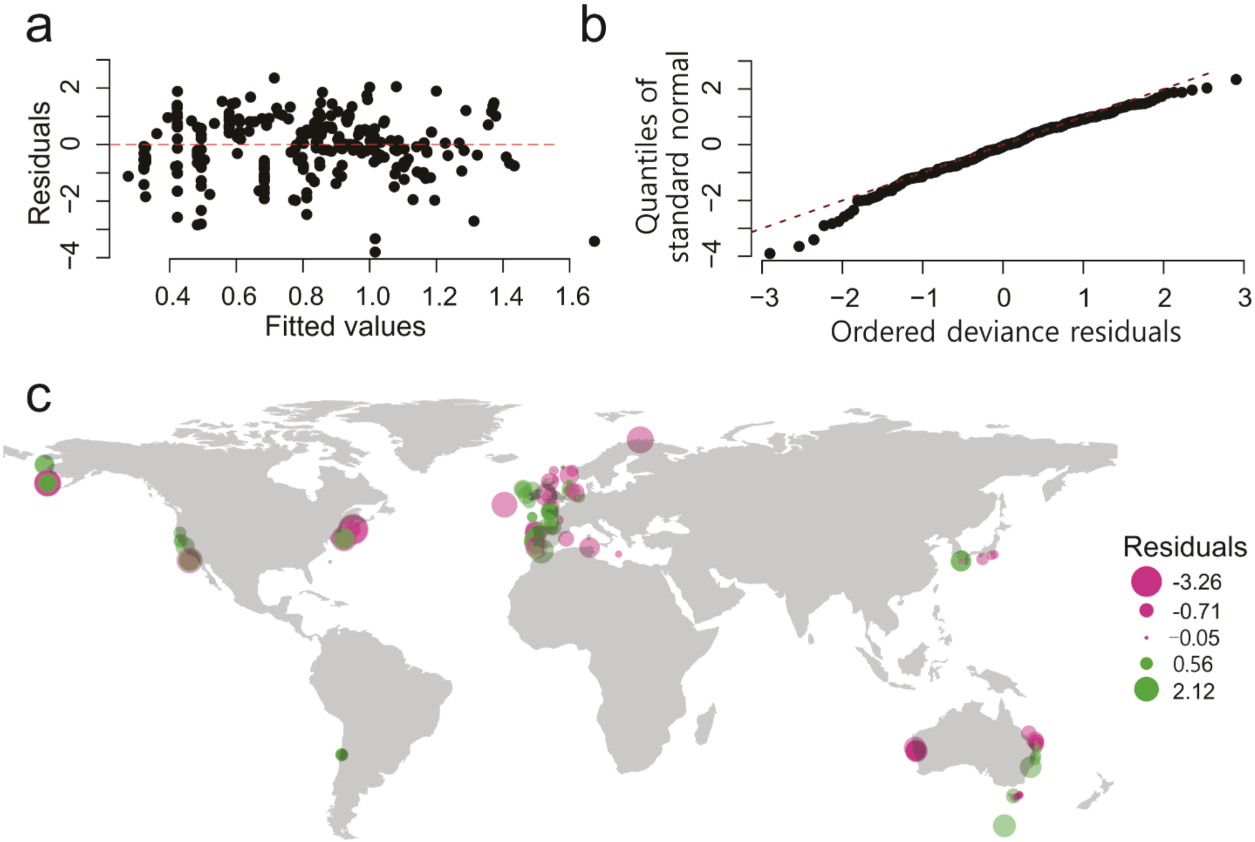

Figure S2. Model diagnostic plots for the most parsimonious model for the prediction of distribution shifts in marine biota. a) Plot of the jackknife deviance residuals against the fitted values, b) normal QQ plot of the standardized deviance residuals, and c) spatial distribution of residuals with dichotomous color scheme for positive/negative values. Size of bubbles proportional to the magnitude with scale set at median, 25/75 percentiles and minimum/maximum residual values. This figure was generated using ArcGIS 10.2 (ESRI, Redland, CA; www.esri.com) and R 3.2.3 (http://www.R-project.org).
